# Deaths of Despair: A Scoping Review on the Social Determinants of Drug Overdose, Alcohol-Related Liver Disease and Suicide

**DOI:** 10.3390/ijerph191912395

**Published:** 2022-09-29

**Authors:** Elisabet Beseran, Juan M. Pericàs, Lucinda Cash-Gibson, Meritxell Ventura-Cots, Keshia M. Pollack Porter, Joan Benach

**Affiliations:** 1 Research Group on Health Inequalities, Environment, and Employment Conditions, Pompeu Fabra University, 08002 Barcelona, Spain; 2 Liver Unit, Internal Medicine Department, Vall d’Hebron University Hospital, Vall d’Hebron Institute for Research, CIBERehd, 08036 Barcelona, Spain; 3 Johns Hopkins University—Pompeu Fabra University Public Policy Center (UPF-BSM), 08002 Barcelona, Spain; 4 UPF Barcelona School of Management, Pompeu Fabra University, 08008 Barcelona, Spain; 5 Lawrence S. Bloomberg Faculty of Nursing, University of Toronto, Toronto, ON M5T 1P8, Canada; 6 Department of Health Policy and Management, Johns Hopkins Bloomberg School of Public Health, Baltimore, MD 21205, USA; 7 Ecological Humanities Research Group (GHECO), Universidad Autónoma de Madrid, 28049 Madrid, Spain

**Keywords:** death of despair, public health, social determinants of health, health inequalities

## Abstract

Background: There is a lack of consensus on the social determinants of Deaths of Despair (DoD), i.e., an increase in mortality attributed to drug overdose, alcohol-related liver disease, and suicide in the United States (USA) during recent years. The objective of this study was to review the scientific literature on DoD with the purpose of identifying relevant social determinants and inequalities related to these mortality trends. Methods: Scoping review focusing on the period 2015–2022 based on PubMed search. Articles were selected according to the following inclusion criteria: published between 1 January 2000 and 31 October 2021; including empirical data; analyzed DoD including the three causes defined by Case and Deaton; analyzed at least one social determinant; written in English; and studied DoD in the USA context only. Studies were excluded if they only analyzed adolescent populations. We synthesized our findings in a narrative report specifically addressing DoD by economic conditions, occupational hazards, educational level, geographical setting, and race/ethnicity. Results: Seventeen studies were included. Overall, findings identify a progressive increase in deaths attributable to suicide, drug overdose, and alcohol-related liver disease in the USA in the last two decades. The literature concerning DoD and social determinants is relatively scarce and some determinants have been barely studied. However different, however, large inequalities have been identified in the manner in which the causes of death embedded in the concept of DoD affect different subpopulations, particularly African American, and Hispanic populations, but blue collar-whites are also significantly impacted. Low socioeconomic position and education levels and working in jobs with high insecurity, unemployment, and living in rural areas were identified as the most relevant social determinants of DoD. Conclusions: There is a need for further research on the structural and intermediate social determinants of DoD and social mechanisms. Intersectional and systemic approaches are needed to better understand and tackle DoD and related inequalities.

## 1. Introduction

Overall, mortality trends were declining in several high-income countries before the COVID-19 pandemic started in 2020 [1], yet a significant increase in excess deaths has been reported across continents since then, which has not only been attributable to SARS-CoV-2 infection but also other causes. In the United States (USA), mortality due to drug overdoses, depressive and mental illness as well as alcohol consumption sharply increased during the COVID-19 lockdown period [2]. However, prior to the pandemic, mortality rates for certain groups of American citizens rose dramatically, mainly due to causes such as suicide, drug overdose, and liver disease due to alcohol abuse [3]. The misuse and addiction to opioids, including the prescription of pain relievers, heroin, and synthetic opioids [4], has been a public health concern for many years in the USA [5].

In 2015, Case and Deaton conducted a pivotal study [3] on the increase in deaths attributable to drug overdose, alcohol liver disease, and suicides between 1999 and 2013. They later labeled this phenomenon *Deaths of Despair* (DoD) [6]. This study was the first to document this reversal of mortality trends after decades and demonstrate this unique phenomenon in the USA. According to Case and Deaton [3], the largest increase in DoD was particularly among middle-aged non-Hispanic whites, especially those with lower levels of education. Between 1999 and 2013, the author calculated that all-cause mortality for less educated non-Hispanic whites increased by 134 per 100,000. Among this group, mortality due to poisonings rose 44.3 per 100,000, chronic liver disease rose 12.2 per 100,000, and intentional self-harm 44.3 per 100,000 [3]. 

Case and Deaton conceptualized despair as having an array of mechanisms causing self-harming behaviors that may underlie a common epidemic of pain and distress and also suggested that the Social Determinants of Health (SDH)—the societal conditions within which people live that shape and influence their health outcomes—play an important role [7].

“[DoD] *come from a long-standing process of cumulative disadvantage for those with less than a college degree. The story is rooted in the labor market, but involves many aspects of life…Although we do not see the supply of opioids as the fundamental factor, the prescription of opioids for chronic pain added fuel to the flames, making the epidemic much worse than it otherwise would have been…Controlling opioids is an obvious priority, as is trying to counter the longer term negative effects of a poor labor market on marriage and child rearing, perhaps through a better safety net….*” [6].

There is consensus amongst researchers that the recent increase in self-harming attitudes such as suicides and drug abuse in the USA have joint characteristics. For instance, mental health issues, such as anxiety disorders, mood disorders, schizophrenia, and other psychotic disorders, are widely recognized as intermediate drivers [8]. At the structural level, a progressive erosion of social relationships, networks, institutions, and communities in large social strata mainly due to economic recessions is also recognized [9]. For instance, exposure to economic stagnation, declining incomes, and prospects as well as exit from the workforce could lead to despair-related behaviors [10]. Few authors have addressed the relationship between such intermediate drivers and the structural determinants of despair-related outcomes. The available evidence suggests a strong role in education attainment, geographical setting, and race/ethnicity [11,12,13]. However, the underlying causes and social mechanisms of DoD and inequalities in DoD-related mortality by social groups seem to be neglected in the current literature. Consequently, there is a lack of consensus among scholars and policymakers as to whether DoD constitutes a distinct epidemiological phenomenon and therefore requires a tailored investigational and interventional approach. Thus, the aim of this study is to review the scientific literature on DoD in the USA published in the health sciences field to identify relevant social determinants.

## 2. Materials and Methods

This scoping review focused on the period from 2015 to the present date (31 April 2022) as DoD was first identified by Case and Deaton in 2015 [3]. A scoping review was chosen due to its exploratory nature and ability to identify and synthesize the evidence on a particular topic that has been limitedly explored [14]. 

### 2.1. Search Strategy

A search on the PubMed database was performed using the following terms and applied to the title, abstract, or keywords: (“deaths of despair” OR “despair premature deaths,” OR “mortality from despair”) AND (“social determinants of health” OR “health inequalities” OR “health disparities” OR “public health”). 

### 2.2. Inclusion and Exclusion Criteria

Studies were eligible for inclusion if they met the following criteria: (1) published between 1 January 2015 and 31 April 2022; (2) empirical (i.e., comments and editorials were excluded); (3) included all DoD categories as defined by Case and Deaton (suicide, acute illicit drug poisoning and alcohol-related deaths) [3,6]; (4) analyzed at least one SDH; (5) written in English; and (6) studied DoD in the USA context only. Studies were further excluded if they met the above inclusion criteria but only analyzed the adolescent population (12–18 years old).

When analyzing deaths due to alcohol, Case and Deaton only included alcohol-related liver disease in DoD [3]; however, we considered alcohol-related deaths in more general terms following the criteria of subsequent authors [8,15,16,17,18,19,20,21]. 

### 2.3. Data Extraction

Articles were screened based on keywords, title, and abstract and then were assessed for full-text reading. Data extracted included study design, study objective, measures used, key findings, and limitations. Two independent reviewers (EB, JMP) selected and analyzed the relevant studies. For each study, the research questions, results, data, and limitations were identified. Findings were synthesized in a narrative report and study results were summarized in tables and qualitatively described.

For the purpose of presenting thematic results in the review, six categories were created for different SDH: (1) economic conditions, referring to socioeconomic insecurity (i.e., income losses, unemployment, and loss of socioeconomic status); (2) occupation and employment conditions, encompassing job hazard risks; (3) education; for which categories were simplified into three: less than high school degree, intermediate and those with more than a college degree, (4) geographical setting, referring to rural vs. urban settings and the location within the USA; (5) race/ethnicity, and (6) gender. To provide an intersectional analysis, which considers multiple axes of inequalities, we also extracted the data about race/ethnicity in each paper included in each category.

### 2.4. Definitions 

Health inequalities refer to unfair, unjust, avoidable, and unnecessary (i.e., neither inevitable nor irremediable) and systematic health differences, within and between countries due to underlying social structures and political, economic, and legal institutions [7]. Socioeconomic position is an aggregate concept that includes both resource-based and prestige-based measures, as linked to both childhood and adult social class position [7]. Job insecurity refers to the concern about whether one will have a job in the future [22]. Economic insecurity is the possibility of a decline in income and socioeconomic position/status [15]_._ Axes of health inequality are inequalities in the social structure due to social class, socioeconomic position, ethnicity, or race that influence the opportunities for having good health [23]. Occupational hazards refer to the characteristic factors of different jobs that may influence despair-related mortality trends [24].

## 3. Results

**Figure 1:**
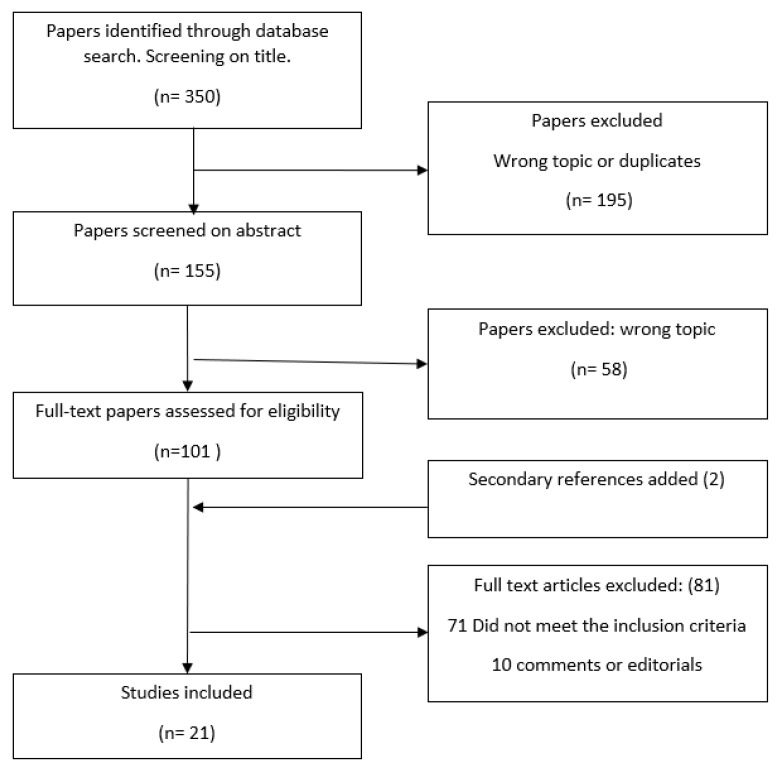
Flowchart of analyzed studies.

A total of 350 articles were identified using the search strategy (Figure 1). The initial screening by title led to the exclusion of duplicates and the initial exclusion of 195 studies that were not in line with the inclusion criteria. A review of abstracts of the remaining 155 resulted in the exclusion of 58 studies that did not address the topic of interest. Hence, 97 full-text articles were read for potential inclusion. A secondary search (i.e., a review of the reference list of identified papers) resulted in two additional studies being included. At the last stage of the review, 81 articles were excluded because the studies did not assess jointly the three potential causes of DoD as a common phenomenon or did not assess any SDH or were editorials or comments, leaving 21 eligible studies.

The first study on DoD was published in 2015 by Case and Deaton [3], two studies were published in 2017 [25,26], and the rest of the literature identified was published during 2019–2022 [5,8,15,16,17,18,19,20,21,24,27,28,29,30,31,32,33,34]. In terms of geography, most of the studies were conducted with data of participants that resided nationwide [3,8,15,16,17,18,20,21,25,29,30,32,34,35]. Only five studies focused on a single USA state [24,27,28,31,33]. All causes of DoD were aggregated in the same cluster of analysis in most of the studies. Few authors separately examined mortality trends for each cause of death, with results suggesting that the main contributor to DoD mortality was opioid-related deaths [24,29,36]. Eleven studies examined race/ethnicity [3,17,19,20,24,25,28,29,30,34,35]. Case and Deaton [3] highlighted that DoD was restricted particularly among those aged 45–64. This finding was supported by subsequent studies which analyzed robust databases [20,25,27,35]. 

### 3.1. Economic Conditions 

Seven studies [8,15,16,17,18,26,33] assessed direct measures of economic conditions related to despair-related outcomes. The results and specific characteristics of each study are shown in Table 1. 

With regards to absolute income, only one study assessing the relationship between the net worth of individuals’ income and the risk of dying due to despair-related causes was found: Zeglin and colleagues [27] analyzed the role of income alongside other SDH in DoD in Florida. Surprisingly, higher than average income (an average income in Florida is 43.000$ per year with a standard deviation of 7.21) was associated with higher mortality due to DoD. Authors hypothesized that “…*perhaps, in counties with above average income, there is lower comparative income utility, thus leading to greater despair and DoD-related behaviors*” [27].

Income insecurity is another factor that has been associated with despair-related mortality. In USA counties, higher economic insecurity has been associated with higher rates of DoD. Knap et al. [16] showed that 20% of USA counties experienced an increase in economic insecurity between 2000 and 2010. Moreover, financial reductions, i.e., loss of assets and loss of income, were also factors that contributed to despair-related mortality [16]. These financial losses were found to mediate 20% of the relation between education with drugs and suicide ideation. This suggests that financial losses among those with lower education levels are an important driver of despair-related behaviors. Conversely, Jou et al. explored housing as another driver of despair mortality [17]. This research found that households that experienced an unexpected positive shock in their housing wealth when they sell their houses had a decreased drug-related mortality rate by 0.27.

Medical coverage was analyzed only in one study [8]. The data were extracted from a Healthcare database with participants that resided nationwide, but most data came from Pennsylvania, West Virginia, and Delaware. Those with the Affordable Care Act or Medicare Coverage were seen to experience respectively 1.3- and 1.5-fold higher risk of suffering DoD relative to those with commercial coverage [8]. The main USA policies that are used to increase income among low-earning workers are Earned Income Tax Credit and Minimum Wages. The use of these policies has been linked to a reduction in non-drug suicides [18].

In summary, our findings show that the main economical drivers related to DoD include a decline in income and assets as well as medical coverage. The perception of worsening social status and economic hardship with respect to close contacts and society overall also seems to have a detrimental effect. Policies that improve economic conditions among low-earning workers appear to be protective against DoD. 

### 3.2. Occupational Hazards

Seven studies analyzed specific occupational risk factors related to despair mortality [24,26,28,30,31,32,33]. Occupational hazards refer to the characteristic factors of different jobs that may influence despair-related mortality trends [24]. Details of the studies are shown in Table 2. 

Hawkins et al. analyzed deaths certificates data in Massachusetts for which the cause of death was a despair-related behavior [28]. The authors found that blue-collar workers exhibited particularly elevated risk for DoD, and among them, those with higher despair-related mortality risk were construction, farming, fishing, and forestry workers [28]. The specific risk for DoD among fishing workers in Massachusetts was supported by Fulmer S et al. study [31]. They found that commercial fishermen were four times more likely to die of opioid poisoning than non-fishermen living in the same fishing ports [31]. When analyzing data for the National Occupation Mortality Surveillance in the USA, Rayhsall et al. found that occupations at higher risk were construction, architects, food, and preparation and service. However, those with the highest increase in DoD were personal care, service, and home aides [32].

Hawkins et al. [24] also studied the contribution of occupation-specific risk factors to DoD in Massachusetts and found that high economic insecurity was one of the most important factors affecting DoD. This economic insecurity was measured as self-reported insecurity by 12% or more of the workers. The occupations which experienced higher economic insecurity were highlighted to be those which suffered higher rates of DoD, particularly opioid-related deaths [24].

Despite the specific risk factors related to each occupation, unemployment and being out of the labor force have been identified as key factors for premature despair-related mortality. Gutin et al. found that managerial/professional administrative groups had an estimated mortality rate of 2.88 per 100,000 individuals compared to 9 deaths/100,000 among unemployed adults, 13.63 deaths among never employed adults, and 19.32 deaths among those not in the labor force [30]. Moreover, this study also showed that there were differences depending on the cause of death among working-aged adults: recent job loss was strongly associated with suicide mortality whereas exit to the labor force was strongly associated with drug poisoning risk [30]. According to Monnat, counties with occupational losses in manufacturing industries are seen to be those with higher deaths of alcohol-, suicide- and drug-related deaths [26].

Under some circumstances, it appears that high-status jobs can also entail a higher risk of DoD. In Gutin et al., health professionals were found to suffer a two-to-four-fold higher accidental poisoning risk relative to adults in managerial/administrative positions. In addition, adults in service exhibited two to three times the risk of mortality from accidental poisoning compared to the same group [30]. Kaki S. et al. studied DoD among healthcare workers in Massachusetts; using death certificated data for 2011–2015, they analyzed the causes of death by occupation. The authors pointed out that the ones that presented higher mortality rates for DoD were medical assistants, nursing, psychiatric, and home health aides; miscellaneous; health technologists and technicians; emergency medical technicians, and paramedics [33].

In summary, employment status and type of employment are factors that were identified to be associated with despair-related mortality. More specifically, although blue-collar jobs exhibited the highest risk of despair-related mortality, not working and specific risk occupation factors lead also to high despair-related behaviors among other occupations apart from blue-collar jobs.

### 3.3. Education Level 

Four studies [3,19,29,34] study the relationship between DoD and educational level. Details of the studies are shown in Table 3. Two studies [16,18] addressing the linkages between income and educational level with regard to DoD are shown in Table 1. 

Educational differences depending on the cause of death had also been reported. Several studies found associations between lower education levels and higher risks of DoD mortality. Case and Deaton reported that DoD was particularly higher among the lesser educated group [3]. In concordance, Zeglin et al. found a significant association between higher-than-average levels of education and a decrease in DoD in Florida [27]. It was found that education disparity was more striking for opioid-related deaths than for suicide or alcohol-related deaths. These differences accounted for 73% and 44%, respectively, for men and women, in drug mortality between low and high education groups [19]. Another study showed that despair-related mortality rates among those with a high-school degree or less were five times higher than those with a college degree, especially for opioid-related deaths [28]. 

As for the relationship between educational level and income, Dow et al. reported that a 10% increase in minimum wage was associated with a 2.7% reduction in suicide deaths for less-educated adults [18]. Moreover, for the earned income tax credit policy, a 10% higher maximum credit reduces suicide by nearly 3%. However, there were no statistically significant effects among the highest education groups [18]. Regarding the role of financial losses, those were seen to be a meaningful mechanism that promotes DoD especially among the lowest educated population. Financial losses accounted for 20% of the association of education with suicide and drug use [16].

Healthcare professionals constitute an exceptional group when considering the causes of DoD and educational attainment since they exhibit one of the highest accidental poisoning and drug overdose risks [29,30]. 

In summary, lower education attainment has been shown to play a crucial role in rising despair mortality trends. However, there is controversy regarding whether this despair-increased mortality is restricted only to the low education level group. The field of work might play a crucial role. 

### 3.4. Geographical Settings 

Three studies analyzed geographical settings [20,25,35]. Details of the studies can be seen in Table 4. 

Stein et al. conducted a nationwide study relying on the Compressed Mortality File, National Center for Health Statistics data for adults aged 25 to 64 years, encompassing 48 subpopulations and two time periods: 1999 to 2001 and 2013 to 2015 [25]. The main findings included an 8% decline in age-adjusted premature death rates for all adults in both sub-periods, with decreases in 39 of the 48 subpopulations. Most decreases in death rates were attributable to HIV, cardiovascular disease, and cancer, whereas most increases in death rates were attributable to suicide, poisoning, and liver disease. All nine subpopulations with increased death rates were non-Hispanic Whites, largely outside large urban areas [24]. However, others reported some differences depending on the cause of death. For instance, in analyzing mortality trends in non-Hispanic White Americans, Monnat found that suicide rates increased among both rural and urban areas, but the increase was larger in the former [23]. Meanwhile, drug poisoning risk was higher in metropolitan areas. Another relevant finding of the study involved large divisional disparities, with particularly poor trends in New England, South Atlantic, East South Central, West South Central, and Appalachia and more favorable trends in the Mid-Atlantic, Mountain, and Pacific [20]. In concordance with these findings, Elo et al. also found that drug poisoning risk was higher in metropolitan areas and that this mortality had a large impact in the Appalachian, East South Central, and New England regions and the smallest in the Pacific region [21].

Regarding economic heterogeneity within rural counties, only one study was found [20]. Mining-dependent counties were found to experience the highest despair-related mortality rates. In contrast, in farming-dependent counties death rates were the lowest. There also were differences depending on the location of the counties, rural southern experienced the highest absolute mortality trends [20]. 

In summary, the geographical setting appears to be a key driver of DoD trends, with rural areas exhibiting the worst despair-related mortality outcomes. Among rural settings, Despair-related mortality inequalities seem to be higher in Southern states in comparison with Northern states. 

### 3.5. Ethnicity/Race

The first study on DoD by Case and Deaton attributed this mortality, especially to non-Hispanic White subgroups [3]. Ulterior studies supported this hypothesis [20,25,28,35]. Notably, several papers only focused their analysis on the non-Hispanic White population group [8,17,21]. Other authors have controlled for race/ethnicity in different studies and did not find significant differences between racial/ethnic groups [16,17,18,19,24,30]. In addition, some other scholars found that DoD was also a concern for other subgroups [20,29]. For instance, Gaydosh et al. showed that despair-related behaviors were increasing among African American, White, and Hispanic populations [29].

In short, there is controversy about the social groups affected by DoD. Even though the phenomenon of DoD was firstly attributed to non-Hispanic Whites exclusively, recent research has pointed out that these trends in mortality are extensible to other ethnic/race subgroups. 

## 4. Discussion

Despite DoD being a relevant phenomenon that has attained a lot of attention in the USA and worldwide, our review revealed that studies on the social determinants of DoD are relatively scarce in health sciences literature. Overall, our findings suggest that DoD can be defined as a cohesive phenomenon that has occurred in the USA since the early 2000s, i.e., there has been a progressive increase in deaths attributable to suicide, drug overdose, and alcohol abuse. In addition, it appears that large inequalities explain how DoD-related causes of death affect different subpopulations. Economic conditions, lower education levels, working in jobs with high insecurity, and being unemployed and living in rural areas appear the most relevant SDH impacting the rates of DoD. Finally, while Case and Deaton claimed that DoD is a phenomenon that essentially affects non-Hispanic Whites [3], several studies have since shown that there are also other ethnic and racial groups that are affected. These findings suggest that public policies should take into account these large social inequalities to prevent DoD among the population. In addition, this study points out the need for further studies to use an intersectional approach to better understand the root causes and social mechanisms of DoD. 

The decline in economic conditions for vast strata of the American population seems to be a major driver of despair-related mortality trends. The deterioration of economic conditions encompasses both objective changes, e.g., lower relative and/or absolute income and socioeconomic position/status, and subjective phenomena such as a perceived frustration with personal and family expectations. The evolution of economic perspectives for the middle class triggered by the aftermath of the 2007–2013 economic crisis has had unequal impacts across Western countries and within countries. Blue collar American workers have generally experienced either stagnation or worsening of economic prospects since the seventies, and therefore frequently share an acute sense of being vanished from the ascending social ladder from which benefited their parents or grandparents [25]. Although other population groups, for instance, African Americans, have the worst absolute mortality outcomes [34], the sense of social status decline in non-Hispanic Whites makes this group more vulnerable to self-harming behaviors driven by increasingly poorer mental health status [34,35,37]. In accordance, previous literature has identified a relationship between lesser perceived social status and depressive symptoms [36,38]. Frustrated expectations might be one of the main mechanisms that promote despair among low and middle-class middle-aged Americans. Remarkably, Zeglin et al. found that in Florida higher income was associated with a higher risk of mortality due to DoD [27]. This unexpected finding highlights the need of considering specific mechanisms and heterogeneity by territory.

The links between economic insecurity and worst mental health, including suicide rates, have been well-known for a long time [39,40,41]. The increase in economic insecurity in the USA, particularly in certain states, was strongly associated with job externalization (e.g., Michigan), the 2007–2013 crisis, and more recently to the COVID-19 pandemic. Burgeoning economic insecurity has led to an epidemic of depression and other mental health issues linked to a sharp increase in suicides and drug-related issues [42].

Occupation, and particularly the type of job, insecurity, and unemployment are also critical factors in the frequency and distribution of despair-related deaths. Precarious employment, characterized by limited financial security, perpetual employment instability, and poor workplace conditions, is progressively affecting greater segments of the American population [43]. Precarious employment is closely linked to anxiety and stress, both precursors of self-damaging behaviors [33], which are also a well-known consequence of unemployment, including suicide and drug overdose [44,45]. Interestingly, some studies suggest that high-status jobs also have increased despair-related mortality. For instance, health professionals exhibit the highest drug mortality risk, which is only partially attributable to easy occupational access to opioids [46].

The greater risk of DoD in the lesser educated population is likely underpinning wider social inequalities. Higher levels of education are known to improve public health and to be protective against poor health outcomes [47]. In contrast, lower levels of education have been associated with a higher risk of suicide and poorer mental health [48]. However, it is unclear if despair is only restricted to the lowest education group or if it is rather a proxy of socioeconomic position as a central axis of inequality that is affected by other social mechanisms, e.g., labor exploitation, discrimination, or racism.

Studies have found that, in general, residents of rural areas experience poorer health outcomes than their urban counterparts [49], and this review suggests that this is also the case for DoD in the USA. Rural communities may have fewer opportunities for gainful employment and experience hardship and difficulties in access to quality health care. Plus, higher isolation and stigmatization of mental illness may promote differences in health behaviors [24]. Moreover, there are widening inequalities across USA states and counties in the incidence of DoD, which underlies the concentration of poorer combinations of social determinants of health in certain American territories.

In the analysis of race and ethnicity, this review encountered diverse results. Some studies found that DoD was higher among White Non-Hispanic Americans. Remarkably, several studies only focused their analysis on this population subgroup [50,51]. Some authors found that other racial groups, for instance, African and Hispanic Americans, were particularly at risk of DoD behaviors. In concordance, Tilstra et al. showed that African Americans exhibited similar despair-related mortality trends to White Americans [52]. These divergent findings suggest the need for additional research into these differences by racial and ethnic groups.

We acknowledge some limitations of this scoping review. First, only papers published in English were included in this review. Second, our review is focused on DoD as a specifically USA phenomenon, thus likely neglecting similar phenomena elsewhere. However, DoD has been also analyzed in other countries [53]. Third, only the PubMed database was searched. Finally, some SDH, such as gender differences were not found because they were little explored in DoD literature, despite differences in alcohol consumption, suicide, and illicit drug behaviors by sex having been reported previously [54,55].

## 5. Conclusions

DoD, i.e., illicit drug poisoning, suicide, and alcohol-related deaths, have sent USA life expectancy falling for several years, making it a national public health concern. Although DoD has been studied more frequently in some USA counties rather than others, is a countrywide epidemic that warrants systemic investigation. Our review found that studies on the social determinants of DoD in the health sciences literature are relatively scarce. Findings from the available literature suggest that the main drivers of DoD and its associated inequalities are the decline in socioeconomic positions and income, as well as rurality and occupation-specific hazards. Low- and middle-income populations and middle-aged Americans are disproportionately affected, and some studies suggest that this is a problem mostly affecting non-Hispanic Whites, although other racial groups such as African Americans and Hispanic seem to be affected too. This review identified a number of gaps in the literature; first, a general lack of intersectional approaches used in the study of DoD and a tendency to concentrate on specific subpopulations, mostly Whites, often neglecting other subpopulations, e.g., African American or Latino women, migrants or homeless persons [56]. Comprehensive studies analyzing the root causes are limited in the currently available health science literature. While some intermediate SDH have been studied (such as unemployment, economic insecurity, and education), others have not been thoroughly addressed (such as access to healthcare), nor have the social mechanisms (such as racism, discrimination, and exploitation) been explored. More research is needed on the structural determinants of despair-related mortality to identify social, physical, economic, and political factors that shape despair-related behaviors and systematically different mortality outcomes, as well as the social mechanisms involved [57]. Intersectional and systemic approaches will also need to be applied. This information can be used to inform the design of effective interventions aiming to reduce DoD and inequalities in DoD outcomes and tackle this national public health crisis.

## Figures and Tables

**Table 1:** Studies addressing Deaths of Despair according to economic conditions.

Author	Year	Aim/s	Methods	Results	Race/ethnicity	Limitations	Sample, Region/State, Year of Data Collection
Brignone E, et al. [8]	2020	To characterize rates of clinically documented DoD over the last decade and identify sociodemographic risk factors.	Retrospective study using healthcare claims database extracted from Highmark, with 10 years of follow-up. Use of logistic regression modeling.	From 2009 to 2018, the prevalence of deaths of alcohol-related, substance-related, and suicide-related diagnoses, respectively, increased by 37%, 94%, and 170% and were associated with higher physical and mental conditions, especially among those with Affordable Care Act or Medicare coverage relative to commercial coverage (Adjusted Odds Ratio (AOR) 1.30, 1.24 to 1.37; AOR 1.51, 1.46 to 1.55). Overall, odds for current-year diagnosis were higher among men (AOR 1.49, 95% CI 1.47 to 1.51).	Details on race/ethnicity were not available for the sample used.	There is an incomplete nature of social determinants of health data in administrative health records.	Healthcare database with 12,144,252 participants. USA. Data was collected from 2009–2018
Knapp EA, et al. [15]	2019	Evaluate the association between changes in economic insecurity and increases in DoD during 2000–2015	Ecological longitudinal study. Measure economic insecurity using indicators from the Census and Federal Reserve Bank in USA counties for the years 2000 and 2010.	Counties in the highest tertile of economic insecurity in 2000 and 2010 had 41% (95% CI: 1.36, 1.47) higher midlife mortality rates at baseline and a rate of increase of 2% more per 5-year period (95% CI: 1.00, 1.03) than counties with stable low economic insecurity. 20% of counties experienced an increase in economic insecurity between 2000 and 2010. The regions experiencing a more acute increase in economic insecurity were the Midwest and South.	The study only estimated associations among all races in the subgroup of counties where non-Hispanic White death rates were not missing. The racial composition did not account for differences.	Deaths were counted only in smaller counties. Approximately 10% of counties had missing data on our primary outcome. The measure of economic insecurity may mask intercounty variation. Some of these changes seen may be due to changes in the coding.	Sample size not available in the USA. Data were collected from 2000–2010
Zeglin RJ, et al. [27]	2019	Determine what country-level social determinants of health are associated with DoD in Florida	Background regression methodology	Higher than average income (*p* = 0.007), median age (*p* < 0.001), and mental health professionals in a Florida county (*p* = 0.027) were associated with significantly higher DoD rates in that county. Higher than average levels of education (*p* = 0.066) and recent checkups in a Florida county (*p* = 0.046) were associated with a significant decrease in DoD in that county. Also, the interaction between income and age was significant in the negative direction (*p* = 0.04) This accounts for 44% of the variance in county level.	Race was not retained in the final model.	The removal of 10% of the counties from analysis due to outliers Small sample size The study did not address assessing for regional effects	Data for all 67 Florida Counties. Florida, USA. Data was collected in 2018.
Fishman SH, et al. [16]	2021	To assess empirically the significance of financial losses as a key mechanism through which education is associated with a higher risk of DoD.	Extract data from the National Longitudinal Study of Adolescent and Adult Health (Add Health). Use of logistic regression modeling.	Financial losses mediate 20% of the association between drug use and suicide ideation among those with those with a high school degree or less. It is possible that other mechanisms operate through financial loss to influence DoD mortality.	Did not report racial differences.	The models do not account for the selection of unobserved characteristics not included in Add Health.The results apply to the association between young adulthood and middle adulthood but may not apply to older ages.	8.000 responders USA Data were collected from 1994–1995 2007–2008 2016–2018
Jou A, et al. [17]	2020	To assess the role of housing as a driver of DoD.	Data were extracted from the Panel Study of Income Dynamics (PSDI).	One Standard Deviation positive shock in housing wealth increases the probability of an improvement in self-reported health by 1.13% points and decreases the drug-related mortality rate by 0.27%. These results are not significant for alcohol or suicide death rates.	Did not find racial differences.	Housing Wealth, Health and DoD	Sample size is not available. USA. Data was collected from 1984–2013.
Dow WH, et al. [18]	2020	To investigate if minimum wages and earn income tax mitigate the rise of DoD	Causal models (difference-in-differences models)	A 10% minimum wage increase reduces non-drug suicides among low-educated adults by 2.7 percent, and the comparable Earned Income Tax Credit figure is 3.0 percent. There are no significant effects on drug- or alcohol-related mortality.	Did not estimate heterogeneity by race or ethnicity.	Potential heterogeneity by race or ethnicity was not estimatedThe models did not pick up long-run effects	Sample size not available. USA. Data was collected from 1999–2017
Pierce R, et al. [35]	2020	To investigate the impact of a large and persistent economic shock on DoD.	Data was extracted from CDC’s National Center for Health Statistics. It provides death certificates from 1990 to 2013. Age-adjusted and crude rates were calculated. Baseline differences specifications to examine the link between deaths of despair and permanent normal trade relations.	Areas more exposed to a plausibly exogenous change in international trade policy exhibit relative increases in fatal drug overdoses (2 to 3 per 100,000), specifically among whites, controlling for state-level legislation pertaining to opioid availability and health care.	The associations between exogenous trade in USA policy and drug overdoses were seen only among whites.	The findings do not provide an assessment of the overall welfare impact of liberalization.	All death certificates in USA. Data were collected from 1990–2013

**Table 2:** Studies addressing Deaths of Despair according to occupational hazards.

Author	Year	Aim/s	Methods	Results	Race/Ethnicity	Limitations	Sample, Region/State, Year Data Collection,
Rayhall, et al. [32]	2022	To assess occupational differences in proportional mortality ratios (PMRs) and trends in these PMRs due to the DoD in the United States	PMRs for deaths due to drug overdoses, suicide, and alcoholic liver disease were obtained from the National Occupational Mortality Surveillance system. Data came from various states for the years 1985 to 1998, 1999, 2003 to 2004, and 2007 to 2014.	Occupations with a higher risk of Deads of Despair were: construction, architects, food preparation, and service. In addition, personal care and home aides had the highest increase in death due to deaths of despair.	Differences between race/ethnicity were not reported.	The occupational codes only consider the occupation of the deceased individual during the majority of their life. It does not consider the occupation of the individual when they passed.	Sample size not available. USA. Data were collected from: 1985–1999 2003–2004 2007–2014
Kaki S et al. [33]	2021	To explore DoD mortality rates among healthcare workers in Massachusetts from 2011 to 2015	Deaths certificated due to DoD were coded by the occupation of the healthcare workers. Mortality rates and rate ratios were calculated according to the occupation of healthcare workers.	The highest mortality rate for DoDs was among medical assistants; nursing, psychiatric, and home health aides; miscellaneous; health technologists and technicians; emergency medical technicians, and paramedics.	Differences between race/ethnicity were not reported,	Misclassification with respect to occupation, as death certificates have only information about the usual occupation of the individual.	Sample size not available Massachusetts, USA. Data were collected from 2011–2015
Fulmer S, et al. [31]	2021	To determine the differences in DoD between fisherman and non-fisherman workers in two Massachusetts fishing ports between 2000–2014	Death certificates were obtained from the Massachusetts Department of Public Health’s Registry of Vital Records and Statistics. The mortality analysis was used to quantify the differences between fishing and non-fishing workers residing in the two cities.	Fishermen were more than four times more likely to die from opioid poisoning than non-fishermen residents.	Racial differences were not reported.	Unique specific fishery characteristics were not determined. It was not possible to differentiate between recreational use and prescription use of opioids.	26,000 deats certificates records Massachusetts, USA 2000–2014
Hawkins D, et al. [24]	2021	To determine whether differences in the risk of DoD were associated with the rate of occupational injuries and illnesses, job insecurity, and temporal changes in employment.	Usual occupation information was collected from death certificates of Massachusetts residents aged 16–64 with relevant causes of death between 2005 and 2015. These data were combined with occupation-level data about occupational injuries and illnesses, job insecurity, and non-standard work arrangements.	The highest number of all deaths of despair occurred in occupations with injury rates of at least 100 per 10,000 full-time workers. Workers in occupations with more job insecurity had higher rates of DoD (RR 1.05 (1.00, 1.09)). Rates of DoD increased most rapidly for occupations with the increasing prevalence of workers employed in non-standard work arrangements (RR 7.6 (6.7, 8.5))	Racial differences were not found	The measures of exposure utilized for this study were assessed at the group level. Within groups, there is likely variation in exposure and individual-level misclassification. The wide variety of data sources that were combined at the national level, certain occupations and workers could have been misclassified with respect to the exposures analyzed. Death certificates only have information about the usual occupation.	Sample size not available Massachusetts, USA Data were collected from 2005–2015.
Hawkins D, et al. [28]	2020	To explore mortality rates and trends according to the occupation of workers who died from DoD	Death certificates for deaths due to poisoning, suicides, and alcoholic liver disease occurring in Massachusetts from 2000 to 2015 were collected and coded according to the occupation of the deceased. Mortality rates and trends in mortality were calculated for each occupation.	DoD increased more than 50% between 2000 and 2004 and 2011–2015. Workers with elevated trends for these deaths were construction, farming, fishing, and forestry workers.	Mortality rates from deaths of despair caused among white, non- Hispanics were about 50% higher than those among Hispanics of all races and black, non-Hispanics. Asian, non-Hispanics accounted for less than 1% of all deaths of despair.	The occupation information listed on a death certificate is the ‘‘usual’’ occupation in an individual’s lifetime. It is not known whether those who died were working at the time of death, which may have resulted in some misclassification of causes of death.	Sample size not available Massachusetts, USA Data were collected from 2000–2015
Gutin I, et al. [30]	2020	To estimate associations between an individual’s occupation and employment status and alcoholic liver disease, suicide, or poisoning mortality risk.	Data of 360,146 adults aged 25–65 from the National Health Interview Survey-Linked mortality Files (1997–2015)	Adults in service jobs (OR 3.10 [1.38–6.94]), manual labor (OR 2.22 [1.00–4.96]), and transport occupations (OR 2.38 [1.08–5.25]) had two to three times the risk of accidental poisoning mortality. Health professionals exhibited the highest accidental poisoning mortality risk (OR 3.35 [1.18–9.47]). Long-term unemployed adults had elevated risk of accidental poisoning (OR 6.44 [2.69–15.41]).Adults not in the labor force had double the suicide risk (2.28 [1.49–3.49]) and seven times the accidental poisoning risk (OR 12.71 [7.46–21.67]).	Racial differences are not found.	Temporal trends were not assessed because of the moderate number of deaths available.Employment status and occupation were obtained at the time of the survey and may change during the follow-up. The accidental poisoning category encompasses a diverse set of deaths. Comprehensive data on individuals’ workplace experiences, exposures, and rewards would allow for a better measure of “precariousness.”	Sample size not available USA. Data were collected from 1997–2015
Monnat [26]	2017	To determine associations between county factors and drug, alcohol, and suicide mortality rates.	Mortality rates were obtained from the U.S Centers for Disease Control and Prevention’s Wide-Ranging Online Data for Epidemiological Research 2006–2015. Spatial analyses were conducted on country-level factors.	Mortality rates are higher among counties with socioeconomically disadvantaged residents, declines in income, military residents, and population aged 65+. Counties with occupational losses experience higher mortality rates due to alcohol, suicide, and drugs.	Racial differences were not reported.	There was no disaggregation by sex or age. Death certificates might misclassify the cause of death. Changes in mortality were not examined.	Sample size not available USA. Data were collected from 2011–2015

**Table 3:** Studies addressing Deaths of Despair according to education level.

Author	Year	Aim/s	Methods	Results	Race/Ethnicity	Limitations	Sample, State/Region, Year Data Collection, Region
Case A, et al. [3]	2015	To analyze the mortality trends in the USA population of all-cause deaths.	Data extracted from CDC Wonder Compressed and Detailed mortality files Ethnicity and educational data were extracted from the American Community Surveys and before 2000 from Popular Surveys.	Over the 15-year period, midlife all-cause mortality fell by more than 200 per 100,000 for black non-Hispanics, and by more than 60 per 100,000 for Hispanics. White non-Hispanic mortality rose by 34 per 100,000 for ages 45–54. The change in all-cause mortality for white non-Hispanics 45–54 is largely accounted for by an increasing death rate in drug and alcohol poisoning and suicide.	There is an increase in non-Hispanic mortality.	No mortality trends analyzed from 2013 onwards.	Sample size not available USA. Data was collected from 1999–2013
Gaydosh L, et al. [29]	2019	To test if the indicators of despairs are rising among middle-aged US adults. To test if this rise is concentrated among low-educated white adults and in rural areas.	Data were extracted from the National Longitudinal Study of Adolescent to Adult Health. The individuals were non-Hispanic White, non-Hispanic Black, or Hispanic. Changes in indicators of despair from adolescence to adulthood were examined using multilevel regression analysis.	Generalized increase in multiple despair indicators among all White, Black, and Hispanic adults in their 30s. Midlife mortality increased across racial/ethnic groups.	There is a general increase in despair across racial/ethnic, educational, and geographical groups.	Indicators may not completely capture all domains of despair. The authors did not examine adults in their 30s because of the lack of data in Add Health sample.	18,446 responders USA. Data were collected from 1994–2017.
Geronimus AT, et al. [19]	2019	To test the empirical evidence of DoD	Using data from Census and Vital Statistics, the authors applied table methods to calculate cause-specific years of life lost between ages 25 and 84 by sex and educational rank for non-Hispanic blacks and whites in 1990 and 2015.	Drug overdose deaths increased over the period 1990 and 2015, particularly in the 25–64 years old group (73% and 44% for men and women, respectively), especially for Whites but trivially for blacks. Both blacks and whites show increased mortality between the lowest and highest education categories.	The contribution of drug overdose deaths increased substantially over the study period for whites, particularly in the 25- to 64-year-old age group.	Deaths due to opioids may be undercounted on death certificates. Deaths may be misclassified.	2,151,890 men and 2,718,198 women USA, not state/region specified. Data were collected from 1990–2015.
Siddiqi A, et al. [34]	2019	To investigate if mortality increases are attributable to (false) perceptions of whites that they are losing social status.	Administrative, survey data, trends, and correlations between race, age, education-specific mortality, and economic and social indicators were examined to determine whether changes in the Republican share of voters during presidential elections were associated with changes in working-age white mortality from 2000 to 2016.	Rising white mortality is not restricted to the lowest education bracket and is occurring deeper into the educational distribution. Parallel trends of economic factors (and more adverse levels) of these factors were being experienced by blacks, whose mortality rates are not rising.	Mortality rates for Blacks are not rising.	It is difficult to completely isolate the influence of Republican vote share because of the interconnectedness of this variable with other socioeconomic conditions.	Sample size not available USA, not state/region specified. Data were collected from 2000–2016.

**Table 4:** Studies addressing Deaths of Despair according to geographical conditions.

Author	Year	Aim/s	Methods	Results	Race/ethnicity	Limitations	Sample, Year State/Region, Data Collection, Region
Monnat SM [20]	2020	To examine metropolitan versus non-metropolitan and intra-non-metropolitan all-cause-specific mortality trends among working age (25–64) non-Hispanic males and females 1990–2018.	Obtain all-cause and cause-specific mortality rates by sex and age group (25–44 and 45–64). Rates are age-adjusted within each 20-year age group using 10-year population counts and weights.	81% of the non-metropolitan mortality death rate increase is due to increases in drugs, alcohol, suicide, and mental/behavioral disorders (DoD).	The non-metropolitan working-age mortality penalty is growing for all ethnic groups and especially for non-Hispanic whites.	The study only examined the white population.The analysis did not adjust for compositional differences.The study only divided two-division and economic types.	9,211,413 deaths in metro counties and 2,465,300 deaths in nonmetro counties USA. Data were collected from 1990–2018.
Stein EM, et al. [25]	2017	To evaluate trends in premature deaths by cause of death, age, race, and urbanization level in the USA.	Calculate cause-specific death rates using Compressed Mortality File, National Center for Health Statistic data for adults 25 to 64 years [two periods 1999–2001 and 2013–2015]. Define 48 subpopulations	Death rates in rural subpopulations for all races/ethnicities increased among those aged 25 to 64 years by 6%, whereas large urban, suburban, and small or medium metropolitan subpopulations had decreases in death rates by 2% to 20%. These disparities were most pronounced in Whites relative to other racial/ethnic subpopulations and among those aged 45 to 54 years. Most increases in death rates were attributable to suicide, poisoning, and liver disease.	Deaths of despair were most pronounced among non-Hispanic whites relative to other racial groups.	The study was limited by the lack of information about the educational and economic status of descendants and the effects of racial/ethnic misclassification.	Sample size not available USA. Data were collected from 1999–2001 and 2013–2015.
Elo IT, et al. [21]	2019	Estimate the contributions of four key age groups to changes in life expectancy at birth between 1990 and 2016 by metropolitan-nonmetropolitan status and region.	Use of 1990–2016 Multiple Cause of Death data files to tabulate deaths by age, sex, race/ethnicity, cause of death, county, and year. These data were combined to estimate age-specific death rates for all causes.	Mortality from drug overdose, suicide, and alcohol-related causes of death increased and contributed to life expectancy reductions across the metropolitan and no-metropolitan categories, especially from a drug overdose.	The study only focused on non-Hispanic groups	Analyses did not adjust for compositional differences. The study focused on Non-Hispanic whites.	Sample size not available USA Data were collected from 1990–2016.

## Data Availability

Data on the search strategy and analyses will be made available upon request.
